# Trauma-induced disturbances in ionized calcium levels correlate parabolically with coagulopathy, transfusion, and mortality: a multicentre cohort analysis from the TraumaRegister DGU^®^

**DOI:** 10.1186/s13054-023-04541-3

**Published:** 2023-07-06

**Authors:** Dries Helsloot, Mark Fitzgerald, Rolf Lefering, Sandra Verelst, Carlo Missant, the TraumaRegister DGU

**Affiliations:** 1 Department of Anaesthesia and Emergency Medicine, AZ Groeninge Hospital, President Kennedylaan 4, 8500 Kortrijk, Belgium; 2 Department of Cardiovascular Sciences, KU Leuven University Campus Kulak, Etienne Sabbelaan 53, Box 7700, 8500 Kortrijk, Belgium; 3 National Trauma Research Institute, Alfred Health and Monash University, Level 4, 89 Commercial Road, Melbourne, VIC 3004 Australia; 4 Trauma Service, The Alfred Hospital, 55 Commercial Road, Melbourne, VIC 3004 Australia; 5 Institute for Research in Operative Medicine (IFOM), Universität Witten/Herdecke, Ostmerheimer Str.200, Haus 38, 51109 Cologne, Germany; 6 Department of Emergency Medicine, UZ Leuven Hospital, Herestraat 49, 3000 Leuven, Belgium; 7 Department of Public Health and Primary Care, KU Leuven University, Herestraat 49, Box 7003, 3000 Leuven, Belgium; 8 Committee on Emergency Medicine, Intensive Care and Trauma Management (Sektion NIS) of the German Trauma Society (DGU), Berlin, Germany

**Keywords:** Trauma, Ionized calcium, Bleeding, Coagulopathy, Transfusion

## Abstract

**Background:**

To which extent trauma- induced disturbances in ionized calcium (iCa2+) levels have a linear relationship with adverse outcomes remains controversial. The goal of this study was to determine the association between the distribution and accompanying characteristics of transfusion-independent iCa2+ levels versus outcome in a large cohort of major trauma patients upon arrival at the emergency department.

**Methods:**

A retrospective observational analysis of the TraumaRegister DGU^®^ (2015–2019) was performed. Adult major trauma patients with direct admission to a European trauma centre were selected as the study cohort. Mortality at 6 h and 24 h, in-hospital mortality, coagulopathy, and need for transfusion were considered as relevant outcome parameters. The distribution of iCa2+ levels upon arrival at the emergency department was calculated in relation to these outcome parameters. Multivariable logistic regression analysis was performed to determine independent associations.

**Results:**

In the TraumaRegister DGU^®^ 30 183 adult major trauma patients were found eligible for inclusion. iCa2+ disturbances affected 16.4% of patients, with hypocalcemia (< 1.10 mmol/l) being more frequent (13.2%) compared to hypercalcemia (≥ 1.30 mmol/l, 3.2%).

Patients with hypo- and hypercalcemia were both more likely (*P* < .001) to have severe injury, shock, acidosis, coagulopathy, transfusion requirement, and haemorrhage as cause of death. Moreover, both groups had significant lower survival rates. All these findings were most distinct in hypercalcemic patients. When adjusting for potential confounders, mortality at 6 h was independently associated with iCa2+ < 0.90 mmol/L (OR 2.69, 95% CI 1.67–4.34; *P* < .001), iCa2+ 1.30–1.39 mmol/L (OR 1.56, 95% CI 1.04–2.32, *P* = 0.030), and iCa2+  ≥ 1.40 mmol/L (OR 2.87, 95% CI 1.57–5.26; *P* < .001). Moreover, an independent relationship was determined for iCa2+ 1.00–1.09 mmol/L with mortality at 24 h (OR 1.25, 95% CI 1.05–1.48; *P* = .0011), and with in-hospital mortality (OR 1.29, 95% CI 1.13–1.47; *P* < .001). Both hypocalcemia < 1.10 mmol/L and hypercalcemia ≥ 1.30 mmol/L had an independent association with coagulopathy and transfusion.

**Conclusions:**

Transfusion-independent iCa2+ levels in major trauma patients upon arrival at the emergency department have a parabolic relationship with coagulopathy, need for transfusion, and mortality. Further research is needed to confirm whether iCa2+ levels change dynamically and are more a reflection of severity of injury and accompanying physiological derangements, rather than an individual parameter that needs to be corrected as such.

**Graphical Abstract:**

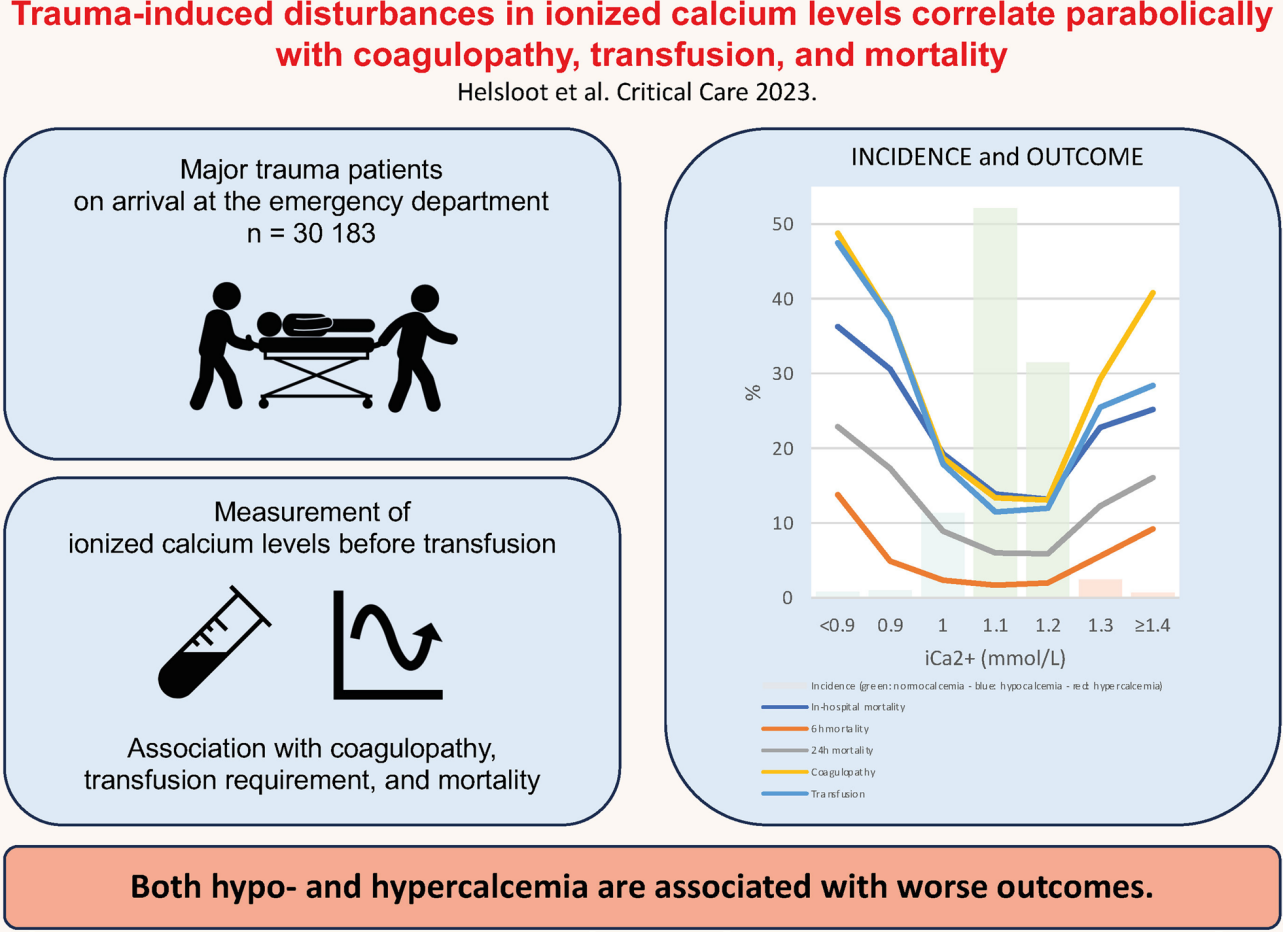

**Supplementary Information:**

The online version contains supplementary material available at 10.1186/s13054-023-04541-3.

## Background

Disturbances in ionized calcium (iCa2+) levels are a frequent finding in critically ill and severely injured patients [1, 2]. It is known that derangements in calcium haemostasis can be life-threatening, but to what extent they contribute to adverse outcome in major trauma patients remains controversial [3, 4].

In contrast to the protein-bound fraction of calcium, the unbound ionized free calcium in the extracellular space is biologically active in a variety of critical physiological pathways including blood coagulation, muscular and cardiac contraction, neuromuscular transmission, and hormone secretion [3]. In major trauma patients most of these pathways are acutely disrupted [1]. Exsanguination causes both loss of calcium ions and disturbance of the calcium homeostasis [5]. Furthermore, massive transfusion protocols using citrate containing blood products further decreases ionized calcium levels as citrate chelates with free calcium, especially in shocked trauma patients who show a decreased hepatic clearance of citrate [6].

The subsequent hypocalcemia can cause disturbances in muscular contractility with decreased cardiac function, vasodilation, and respiratory failure [7–10]. Furthermore, a lack of ionized calcium likely worsens trauma-induced coagulopathy (TIC) [11]. TIC contributes to uncontrolled haemorrhage, which is considered the most common preventable cause of death after major trauma [12].

Previous research has indicated that transfusion-related hypocalcemia during trauma resuscitation is associated with increased mortality, coagulopathy, and blood transfusion requirements [6, 13–17]. Similar associations were found in transfusion-independent hypocalcemia on admission, as demonstrated in a recent systematic review by Vasudeva et al. (2021) [1, 3, 18, 19].

However, most of these findings are reported in small cohort studies, with only moderate evidence [1]. The question remains whether hypocalcemia is the cause of adverse outcome or only a predictive marker. Furthermore, most studies have focused on hypocalcemia, while the incidence and effects of hypercalcemia have been somewhat overlooked. In this regard, a significant risk may occur if empirical or aggressive calcium supplementation would be considered.

The aim of this study was to determine the association between the distribution and accompanying characteristics of transfusion-independent ionized calcium levels versus outcome in a large European cohort of major trauma patients upon arrival at the emergency department (ED).

## Methods

### Study design and data source

For this retrospective observational analysis, data was retrieved from the TraumaRegister DGU^®^.

The TraumaRegister DGU^®^ of the German Trauma Society (Deutsche Gesellschaft für Unfallchirurgie, DGU) is a multicentre database of pseudonymized and standardized documentation of severely injured patients. The inclusion criteria are ED admission to hospital, with life signs on arrival, and subsequent intensive care unit (ICU) management or reaching hospital with vital signs but dying before ICU admission. The participating hospitals are primarily located in Germany (90%), but an increasing number of hospitals from other countries contribute data as well. The ‘full dataset’ is obligatory for all supra-regional trauma centres, only hospitals certified as a regional or local trauma centre within the TraumaNetzwerk DGU^®^ are allowed to complete a ‘basic dataset’.

Laboratory values on admission are defined as the first documented values at time of first blood collection upon arrival at the ED.

A detailed description of the dataset is provided in Additional file 1.

The present study is in line with the publication guidelines of the TraumaRegister DGU^®^ and is registered under the TR-DGU project ID 2020-030.

### Participants

**Fig. 1:**
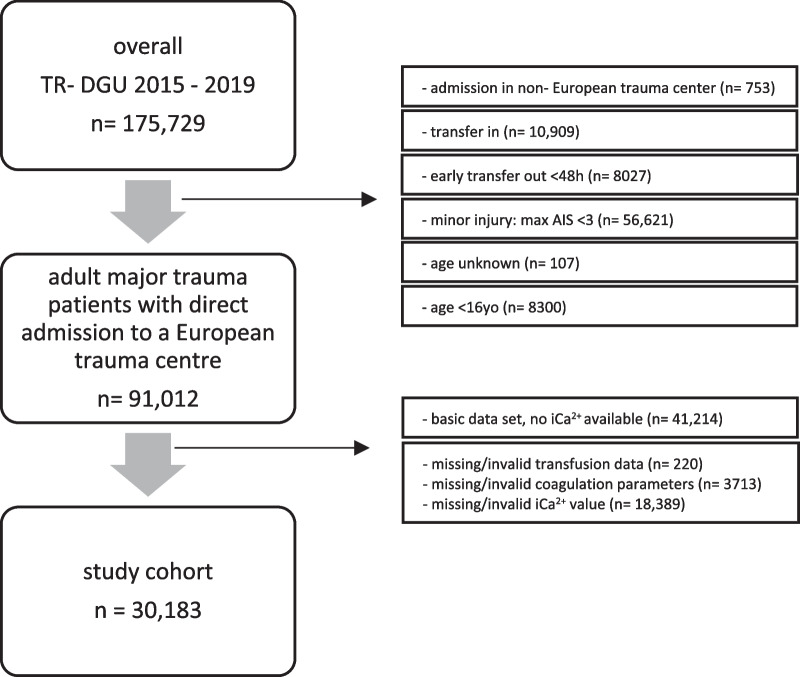
Selection of study sample

The study cohort was selected from the TraumaRegister DGU^®^ ‘version 2015’ dataset (accident years 2015 until 2019). The selection of the study sample is shown in Fig. 1. Adults (≥ 16 years old) with major traumatic injuries (Abbreviated Injury Scale [AIS] ≥ 3) and direct admission from the accident scene to a European trauma centre were considered eligible. Patients who were transferred out to another hospital within 48 h were excluded, due to missing outcome data. Patients with ‘basic dataset’ registration had to be excluded because no iCa2+ levels were recorded in this dataset. When data for transfusion, coagulation, or iCa2+ levels were missing or considered invalid (iCa2+  < 0.3 mmol/L), these patients were also excluded.

To check for potential bias, additional analysis of demographic and outcome data was performed for patients who were excluded because of missing/invalid iCa2+ levels.

### Analysis

#### Distribution, demographics, and outcome analysis

The distribution of documented ionized calcium levels was analysed by measuring the proportion of each level in the study cohort. Ionized calcium levels were categorized as (mmol/l) < 0.9 (< 0.90), 0.9 (0.90–0.99), 1.0 (1.00–1.09), 1.1 (1.10–1.19), 1.2 (1.20–1.29), 1.3 (1.30–1.39), and ≥ 1.4 (≥ 1.40).

For each of these levels, mortality at 6 h, 24 h, and in-hospital mortality was determined. Furthermore, the incidence of coagulopathy upon arrival and transfusion requirement were determined. Coagulopathy was defined as a partial thromboplastin time (PTT) ≥ 40 s or an international normalized ratio (INR) ≥ 1.4, according to the Berlin definition of polytrauma [20]. Transfusion requirement included all transfusions which were initiated during ED admission and/or during emergency surgery prior to ICU admission.

Next, three subgroups were defined, based on the incidence of adverse outcome and in line with previous reports on this topic: normocalcemia (1.10–1.29 mmol/l), hypocalcemia (< 1.10 mmol/l), and hypercalcemia (≥ 1.30 mmol/l) [21].

Relevant patient demographics, clinical characteristics and outcome parameters were retrieved from the registry for further analysis. Continuous variables were recorded as means with standard deviation (SD) if approximal normally distributed and median with interquartile range (IQR) otherwise. Categorical variables were presented as numbers with percentages. Differences were assessed by Student’s t test/Mann–Whitney U-test for continuous and Chi-squared test for categorical variables. A *P* value ≤ 0.05 was considered as statistically significant. However, in large samples small differences could become statistically significant, even without any clinical importance. Therefore, *P* values are not shown in the presentation of the demographics and clinical characteristics in Table 1, as they do not necessarily reflect clinical relevance.

**Table 1:** Relevant demographics and clinical characteristics for different ranges of ionized calcium levels

Variables	Overall	HYPOcalcemia (< 1.10 mmol/L)	NORMOcalcemia (1.10–1.29 mmol/L)	HYPERcalcemia (≥ 1.30 mmol/L)
Total (%)	30,183 (100)	3982 (13.2)	25,238 (83.6)	963 (3.2)
Patient				
Sex, male (%)	21,655 (71.7)	2933 (71.1)	18,160 (72.0)	662 (68.7)
Age, median (IQR)	54 (35–70)	54 (37–70)	54 (35–70)	55 (33–72)
Anticoagulation therapy before accident (%)	4700 (15.6)	595 (14.9)	3953 (15.7)	152 (15.8)
Accident mechanism				
Blunt (%)	27,872 (95.5)	3563 (93.4)	23,436 (95.9)	873 (94.1)
Injury severity				
ISS, median (IQR)	21 (14–29)	22 (16–29)	20 (14–29)	22 (16–29)
Pre- clinical vital signs				
sBP ≤ 90 mmHg (%)	2791 (10.5)	512 (15.0)	2125 (9.5)	154 (18.2)
Shock Index ≥ 1 (%)	3478 (13.5)	587 (18.1)	2746 (12.7)	145 (18.1)
GCS ≤ 8 (%)	7032 (24.7)	1237 (33.1)	5532 (23.2)	263 (28.7)
Pre- clinical therapy				
Volume administration > 1000 ml (%)	4189 (15.2)	548 (15.5)	3456 (14.9)	185 (20.8)
Intubation (%)	9757 (33.1)	1518 (39.5)	7846 (31.8)	393 (41.4)
TXA (%)	4430 (15.0)	672 (17.5)	3593 (14.6)	165 (17.4)
Cathecholamines (%)	3269 (11.1)	567 (14.7)	2514 (10.2)	188 (19.8)
Vital signs on admission				
sBP ≤ 90 mmHg (%)	3072 (10.6)	588 (15.5)	2301 (9.4)	183 (19.8)
Shock Index ≥ 1 (%)	3860 (13.5)	691 (18.7)	2976 (12.4)	193 (21.7)
Temperature < 35 °C (%)	2089 (10.8)	386 (16.1)	1600 (9.8)	103 (16.6)
Laboratory values on admission				
Hb, g/dL, mean (SD)	12.87 (2.22)	12.30 (2.58)	13.01 (2.10)	11.73 (2.77)
Coagulopathy (PTT ≥ 40 s and/or INR 1.4) (%)	4532 (15.0)	873 (21.9)	3352 (13.3)	307 (31.9)
Base Excess, mean (SD)	− 2.20 (4.77)	− 3.65 (6.09)	− 1.82 (4.28)	− 6.19 (7.28)
Acidosis, BE < − 6 (%)	4555 (15.2)	997 (25.3)	3180 (12.7)	378 (40.1)
Transfusion prior to ICU admission				
Transfusion (%)	4042 (13.4)	843 (21.2)	2947 (11.7)	252 (26.2)
Massive transfusion ≥ 10 PRC (%)	528 (1.7)	149 (3.7)	335 (1.3)	44 (4.6)
Procoagulants prior to ICU admission				
Ca2 + supplement (%)	1593 (5.6)	372 (10.4)	1120 (4.7)	101 (11.2)
TXA (%)	5996 (21.2)	1032 (28.9)	4693 (19.7)	271 (29.9)
Length-of-stay				
ICU days, median (IQR)	3 (1–11)	4 (2–12)	3 (1–10)	4 (1–13)
Hospital days, median (IQR)	14 (7–24)	14 (6–26)	14 (7–24)	14 (6–25)
Mortality				
In-hospital mortality (%)	4500 (14.9)	843 (21.2)	3432 (13.6)	225 (23.4)
Mortality < 1 h (%)	131 (0.4)	30 (0.8)	82 (0.3)	19 (2.0)
Mortality < 24 h (%)	2051 (6.8)	413 (10.4)	1511 (6.0)	127 (13.2)
Cause of death				
Hemorraghe (%)	283 (6.4)	88 (10.7)	166 (4.9)	29 (13.1)
Traumatic Brain Injury (%)	2653 (60.4)	451 (55.1)	2098 (62.5)	104 (47.1)
Organ failure (%)	1039 (23.6)	205 (25.0)	761 (22.7)	73 (33.0)
Other (%)	421 (9.6)	75 (9.2)	331 (9.9)	15 (6.8)

*ISS* Injury Severity Score, *AIS* Abbreviated Injury Scale, *GCS* Glasgow Coma Scale, *TXA* tranexamic acid, *Hb* hemoglobin, *PTT* partial thromboplastin time, *INR* International Normalized Ratio, *BE* base excess, *PRC*: packed red cells, *Ca2+*: calcium, *ICU*: intensive care unit, *IQR*: interquartile range, *SD*: standard deviation

Kaplan–Meier survival curves were used to compare differences in time to death, and differences were evaluated with a log rank test.

#### Multivariable logistic regression

Multivariable models have been calculated for mortality (6 h, 24 h, and in-hospital mortality), coagulopathy, and blood transfusion as dependent endpoints. The categorical variable iCa2+ 1.1 mmol/L was considered as the reference group.

From the about 150 variables in the registry, potential predictors were selected a priori to the analysis. Because of the large sample size, statistical significance did not always correlate with clinical significance. Therefore, these variables were chosen based on previous literature studying prediction models for mortality, coagulopathy, and blood transfusion in major trauma patients. Moreover, prior expert discussion and the availability of the data within the registry were considered in the selection of these predictors.

For prediction of blood transfusion and coagulopathy, the following independent predictors were considered: age, male sex, anticoagulation therapy before the accident, blunt trauma, relevant injuries (AIS ≥ 3) in different body regions, Injury Severity Score (ISS), prehospital Glasgow Coma Scale (GCS) ≤ 8, prehospital systolic blood pressure ≤ 90 mmHg, prehospital volume administration of more than 1000 ml. Age was considered in 4 subgroups (16–59 years, 60–69 years, 70–79 years, ≥ 80 years), identical to previous studies, due to the known non-linear effect on mortality. Sample size above 59 years was calculated to ensure it was still large enough to yield statistically stable results [22]. All the above predictors have previously been demonstrated as being relevant for coagulopathy and transfusion [22–28].

For mortality prediction, the Revised Injury Severity Classification II (RISC II) score was used as a summary of 13 known predictors. The year of accident, level of care of the trauma centre, and type of transportation (ground or helicopter) were additionally included for mortality analysis [22, 29]. The RISC score has been used for outcome adjustment in the TraumaRegister DGU^®^ since 2003 and was updated in 2014 to a second version (RISC II). This model predicts hospital mortality after trauma and was repeatedly validated in the years after its introduction. The current model consists of the following predictors: worst and second-worst injury (AIS severity level), head injury, age, sex, pupil reactivity and size, pre-injury health status, blood pressure, acidosis (base excess), coagulation (INR), haemoglobin, and cardiopulmonary resuscitation [29, 30].

In the multivariable models, missing values were included as a separate category.

Nagelkerke`s *R*^2^ was calculated for each model. Adjusted Odds Ratios (OR) were reported with 95% confidence intervals. A *P* value ≤ 0.05 was considered as statistically significant.

Statistical analysis was conducted with SPSS^®^ Statistics software (IBM Corp. Version 27. Armonk, NY, USA). Graphs were created with MS Excel^®^ (Excel for Mac, Version 16.46. Redmond, WA: Microsoft Corp).

## Results

### Study cohort selection

In the TraumaRegister DGU® 175 729 patients were registered within the ‘version 2015’ dataset (2015 until 2019). Of these, 30 183 adult major trauma patients with direct admission to a European trauma centre and full dataset documentation were considered as the study cohort (Fig. 1).

From the 8027 patients who were transferred out within 48 h, only 836 would have met the other inclusion criteria (2.7% of study cohort).

A total of 18 389 patients were excluded because of missing/invalid iCa2 + values. This group had a median age of 55 years, but with lower injury severity (ISS 17) and mortality (13%, RISC II 12.4%) compared to the study cohort.

### Ionized calcium levels and patients’ outcomes

**Fig. 2:**
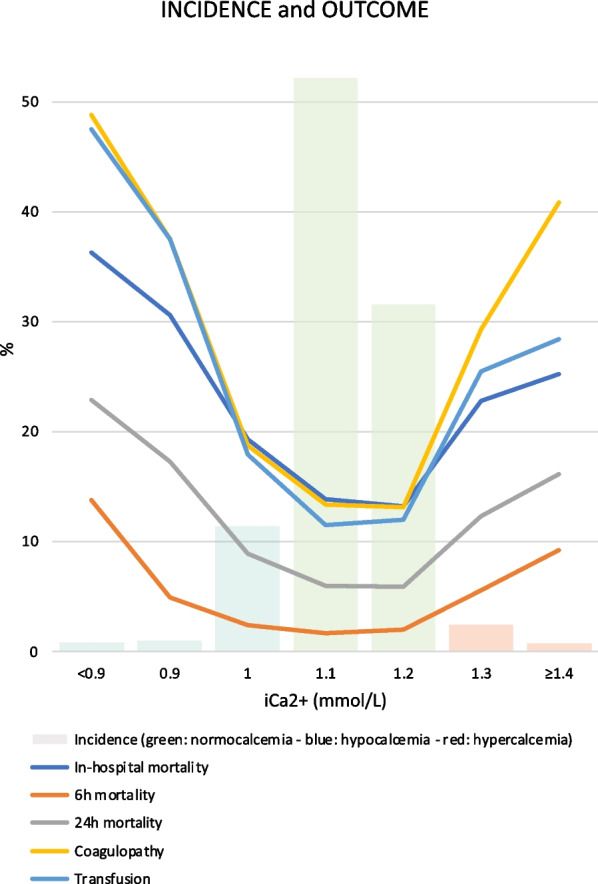
Distribution and accompanying outcome parameters of ionized calcium levels in adult major trauma patients

The distribution of iCa2+ levels on admission is shown in Fig. 2 and Additional file 2. Most patients were normocalcemic upon arrival (n = 25 238, 83.6%). Hypocalcemia was documented in 3 982 patients (13.2%), whereas hypercalcemia was observed in the remaining 963 patients (3.2%).

In the overall study cohort, the recorded in-hospital mortality rate was 14.9%, of which 2.1% died within the first 6 h after admission and 6.8% died within the first 24 h. Coagulopathy was documented in 15.0% of the patients, and 13.4% received transfusion prior to ICU admission. As shown in Fig. 2, all outcome parameters had a similar parabolic distribution, with decreasing and increasing iCa2% levels being associated with increasing incidence of adverse outcome. The highest recorded incidence rates for these parameters were systematically noted in the lowest iCa2+ level, iCa2+  < 0.9 mmol/L (36.3% in-hospital mortality, 48.8% coagulopathy, and 47.5% transfusion).

**Fig. 3:**
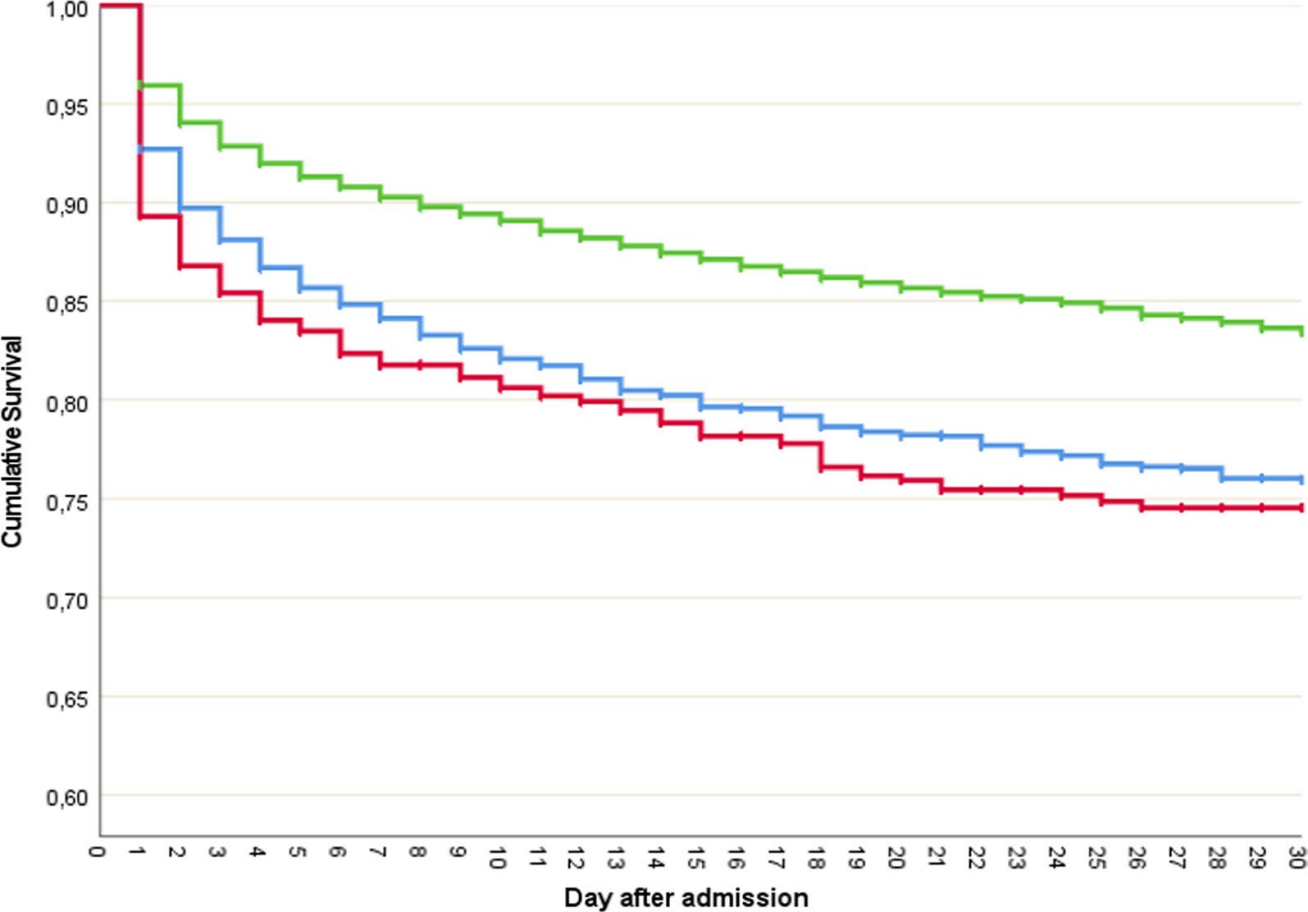
Kaplan–Meier survival curve for the first 30 days after hospital admission. Green line: normocalcemia (1.10–1.29 mmol/L) – blue line: hypocalcemia (< 1.10 mmol/L) – red line: hypercalcemia (≥ 1.30 mmol/L)

A Kaplan–Meier survival analysis revealed lower survival rates in both hypo- and hypercalcemia groups, with the lowest survival in hypercalcemic patients (Fig. 3). Both curves were significantly different from the normocalcemia group but did not show a significant difference among each other (*P* = 0.094). The difference in survival between these groups was mainly due to the differences in early mortality within the first day.

### Demographics and clinical characteristics

Relevant patient demographics and clinical characteristics, according to initial iCa2 + levels, are provided in Table 1 and e-Table 1 (Additional file  3).

Similar characteristics were observed between all groups for sex (male), median age (54 years old), accident mechanism (blunt), anticoagulation therapy before the accident, and median length of in-hospital stay (14 days).

Compared to the normocalcemia group, patients with hypo- and hypercalcemia were more likely to have severe injury (median ISS), shock (shock index [SI] ≥ 1), GCS ≤ 8, and hypothermia (temperature < 35 °C). Laboratory tests revealed lower haemoglobin levels and a higher incidence of coagulopathy and acidosis (base excess < − 6). During pre-hospital resuscitation, both groups were more likely to get intubated and to receive catecholamines. Prior to ICU admission, there was a higher incidence of transfusion, massive transfusion (packed red blood cells ≥ 10units), and Ca2+ supplementation. All these findings were statistically significant (*P* < 0.001) and considered clinically relevant.

Within the abnormal ionized calcium level groups, the hypercalcemic group had more distinct findings for all the above-described parameters when compared to the hypocalcemia group, except for prehospital GCS ≤ 8. This discrepancy was mainly prominent for coagulopathy (31.9% vs. 21.9%) and acidosis (40.1% vs. 25.3%). Compared to normocalcemia, the incidence of prehospital volume administration > 1000 ml was significantly higher for the hypercalcemia group (*P* < 0.001), but not for the hypocalcemia group (*P* = 0.088).

Haemorrhage as cause of death was more likely to occur in the hypercalcemia (13.1%) and hypocalcemia (10.7%) groups compared to the normocalcemia (4.9%) group (*P* < 0.001).

### Multivariable logistic regression

The results of the multivariable logistic regression analysis for mortality (6 h, 24 h, and in-hospital), coagulopathy, and transfusion are presented in Table 2.

**Table 2:** Results of the multivariable logistic regression analysis for mortality (6 h, 24 h, in-hospital), coagulopathy, and transfusion

Variable (reference)	6 h mortality			24 h mortality			In- hospital mortality		
	Adj OR	95% CI	*P* value	Adj OR	95% CI	*P* value	Adj OR	95% CI	*P* value
iCa2+ level (1.1 mmol/L)									
iCa2+ < 0.9	2.69	1.67–4.34	< 0.001	1.12	0.73–1.72	0.60	0.81	0.53–1.22	0.31
iCa2+ 0.9	0.89	0.47–1.67	0.70	1.21	0.80–1.83	0.36	1.07	0.74–1.56	0.71
iCa2+ 1.0	1.06	0.80–1.41	0.68	1.25	1.05–1.48	0.011	1.29	1.13–1.47	< 0.001
iCa2+ 1.2	1.09	0.88–1.34	0.46	0.91	0.79–1.04	0.17	0.96	0.86–1.06	0.39
iCa2+ 1.3	1.56	1.04–2.32	0.030	0.96	0.70–1.32	0.81	0.94	0.72–1.22	0.62
iCa2+ ≥ 1.4	2.87	1.57–5.26	< 0.001	1.71	0.85–2.43	0.17	0.95	0.62–1.56	0.95
RISC II score									
Per point	0.53	0.52–0.55	< 0.001	0.47	0.45–0.48	< 0.001	0.40	0.39–0.41	< 0.001
Level of care (Level 1)									
Level 2	1.19	0.87–1.61	0.27	1.11	0.91–1.35	0.33	1.03	0.89–1.20	0.69
Level 3	1.98	0.85–4.62	0.12	1.45	0.81–2.59	0.21	0.83	0.52–1.32	0.42
Year of accident (2015)									
2016	0.88	0.57–1.33	0.54	1.10	0.84–1.46	0.48	0.93	0.75–1.14	0.47
2017	1.05	0.70–1.58	0.81	0.97	0.74–1.28	0.84	0.90	0.73–1.11	0.31
2018	0.81	0.53–1.23	0.33	0.93	0.71–1.23	0.63	0.94	0.76–1.16	0.55
2019	0.94	0.62–1.43	0.79	1.23	0.93–1.62	0.14	1.16	0.94–1.42	0.17
Transport (ground)									
Helicopter	0.67	0.54–0.82	< 0.001	0.68	0.59–0.78	< 0.001	0.66	0.60–0.73	< 0.001
Unknown	0.79	0.45–1.39	0.41	0.77	0.54–1.10	0.14	1.06	0.83–1.35	0.66

Variable (reference)	Coagulopathy			Transfusion					
	Adj OR	95% CI	*P* value	Adj OR	95% CI	*P* value			
Ca2+ level (1.1 mmol/L)									
iCa2+ < 0.9	4.66	3.45–6.31	< 0.001	4.24	3.07–5.86	< 0.001			
iCa2+ 0.9	3.10	2.36–7.07	< 0.001	3.46	2.58–4.64	< 0.001			
iCa2+ 1.0	1.39	1.25–1.55	< 0.001	1.49	1.32–1.67	< 0.001			
iCa2+ 1.2	0.98	0.90–1.06	0.62	0.96	0.87–1.05	0.33			
iCa2+ 1.3	2.42	2.01–2.92	< 0.001	2.06	1.68–2.53	< 0.001			
iCa2+ ≥ 1.4	4.72	3.45–6.46	< 0.001	2.39	1.66–3.44	< 0.001			
Age (16–59 year)									
60–69	0.98	0.87–1.09	0.67	1.14	1.01–1.28	0.032			
70–79	1.48	1.32–1.65	< 0.001	1.16	1.02–1.32	0.022			
≥ 80	2.04	1.82–2.28	< 0.001	0.96	0.83–1.11	0.57			
Sex (female)									
Male	1.08	1.00–1.17	0.054	0.78	0.72–0.85	< 0.001			
Anticoagulation therapy before the accident (none)									
Anticoagulant drugs	5.00	4.51–5.53	< 0.001	1.21	1.06–1.38	0.004			
Unknown	1.46	1.33–1.60	< 0.001	1.22	1.11–1.34	< 0.001			
Mechanism (blunt)									
Penetrating	1.62	1.37–1.91	< 0.001	3.33	2.87–3.87	< 0.001			
Relevant injury AIS ≥ 3									
Head	1.05	0.95–1.16	0.376	0.70	0.62–0.78	< 0.001			
Thorax	1.11	1.02–1.21	0.018	1.09	1.00–1.19	0.054			
Abdomen	1.52	1.36–1.69	< 0.001	2.67	2.42–2.95	< 0.001			
Extremities	1.45	1.33–1.59	< 0.001	3.02	2.76–3.29	< 0.001			
Injury Severity Score									
ISS, per point	1.035	1.031–1.039	< 0.001	1.050	1.046–1.054	< 0.001			
Prehospital data									
GCS ≤ 8	2.13	1.95–2.32	< 0.001	1.62	1.46–1.79	< 0.001			
sBP ≤ 90 mmHg	2.15	1.94–2.38	< 0.001	2.48	2.23–2.76	< 0.001			
Missing sBP	1.27	1.15–1.41	< 0.001	1.91	1.72–2.13	< 0.001			
Volume > 1000 ml	1.79	1.63–1.97	< 0.001	2.14	1.95–2.34	< 0.001			

Nagelkerke’s R^2^: 6 h mortality: 0.40, 24 h mortality: 0.50, in-hospital mortality 0.58

Nagelkerke’s R^2^: coagulopathy: 0.27, transfusion 0.33

*iCa2 + * ionized calcium, *RISC-II* Revised Injury Severity Classification, version II, *AIS* Abbreviated Injury Scale, *ISS* Injury Severity Score, *GCS* Glasgow Coma Scale, *sBP* systolic blood pressure, *adj OR* adjusted odds ratio, *CI* confidence interval

When adjusting for potential confounders, mortality at 6 h was independently associated with iCa2+  < 0.90 mmol/L (OR 2.69, 95% CI 1.67–4.34; *P* < 0.001), iCa2+ 1.30–1.39 mmol/L (OR 1.56, 95% CI 1.04–2.32, *P* = 0.030), and iCa2+  ≥ 1.40 mmol/L (OR 2.87, 95% CI 1.57–5.26; *P* < 0.001). Moreover, an independent relationship was determined for iCa2+ 1.00–1.09 mmol/L with mortality at 24 h (OR 1.25, 95% CI 1.05–1.48; *P* = 0.0011), and with in-hospital mortality (OR 1.29, 95% CI 1.13–1.47; *P* < 0.001).

For both hypocalcemia < 1.10 mmol/L and hypercalcemia ≥ 1.30 mmol/L an independent association with coagulopathy and transfusion was demonstrated.

## Discussion

This large multicentre analysis of major trauma patients revealed that both low and high transfusion- independent ionized calcium levels on arrival at the ED were associated with increased 6 h, 24 h, and in-hospital mortality. To the best of our knowledge, this study is the first to present the parabolic relationship between ionized calcium levels and mortality. The similar relationship with coagulopathy and need for transfusion supports the hypothesis that iCa2 + disturbances are associated with trauma-induced coagulopathy, causing increased need for transfusion and eventually death [13].

### Incidence

Ionized calcium disturbances affected 16.4% of adult patients with major trauma upon arrival at the ED, with hypocalcemia being more frequent (13.2%) than hypercalcemia (3.2%). In this large European cohort, the reported incidence of hypocalcemia was remarkably lower compared to previous studies, with hypocalcemia being as frequent as 23% up to 74% [1].

Reports on hypercalcemia are sparse because most studies seem to include hypercalcemia in the normocalcemia group. However, MacKay et al. [16] identified 22% of trauma patients with hypercalcemia during trauma resuscitation with high-volume transfusion. Regardless of the low incidence of hypercalcemia in the present analysis, it was also associated with adverse outcomes. Therefore, hypercalcemia cannot be considered a physiologically normal state and should be investigated in a separate group.

The differences in reported hypo- and hypercalcemia levels compared to those in previous studies are likely related to the different selection criteria used [3, 6, 16, 19, 31]. For this analysis, the first measured iCa2+ value on arrival of all severely injured patients was considered, to specifically evaluate the trauma-related effect on calcium haemostasis before transfusion was initiated or extensive resuscitation measures were taken.

### Trauma related hypocalcemia and outcome

It is well known that calcium levels may decrease rapidly during massive transfusion of large volumes of citrate containing blood products [32]. However, as confirmed in this analysis, even upon arrival at the ED calcium disturbances are a common finding before extensive resuscitation and transfusion. This trauma-related hypocalcemia is caused by bleeding-related losses, impaired calcium haemostasis, and increased sympathetic activity [21]. Matthay et al. [18] demonstrated an independent association between initial pretransfusion calcium levels in injured patients and ex-vivo platelet activation, platelet aggregation, and platelet dependent visco-elastic clot-formation, resulting in a significant effect on blood product transfusion. It is believed that this measured ex-vivo platelet activation and aggregation can be explained by the in-vivo calcium-induced priming of platelets by the increase of platelet surface receptors [18].

In contrast to a recent retrospective cohort study by Chanthima, in which the first measured iCa2+ nor the administered calcium dose for citrate load correction were significantly associated with in-hospital mortality, different previous studies have demonstrated an association between hypocalcemia and mortality [1, 4, 13]. A systematic review by Vasudeva [1] revealed an independent association between transfusion-independent hypocalcemia and mortality, coagulopathy, and transfusion of blood products. However, this finding was only of moderate evidence since only three smaller cohort studies were found eligible for inclusion. The present large cohort study confirmed the independent association between low ionized calcium levels on arrival at the ED (< 1.1 mmol/L) and an increased incidence of coagulopathy and need for transfusion prior to ICU admission. Mortality at 6 h, at 24 h, and in-hospital mortality increased in the presence of hypocalcemia. Specifically, death caused by haemorrhage was double in the hypocalcemia group (10.7%) compared to the normocalcemia group (4.9%). Kaplan–Meier analysis confirmed lower survival rates when hypocalcemia was present upon arrival. In line with previous findings by Vasudeva et al. (2020), these lower survival rates were mainly related to increased early mortality [31]. However, in this analysis, an independent association between hypocalcemia and early mortality could only be demonstrated for mortality at 6 h with severe hypocalcemia (iCa2+ < 0.90 mmol/L) and for mortality at 24 h with mild hypocalcemia (iCa2+ 1.00–1.09 mmol/L).

### Is there a role for empirical calcium administration?

Based on these findings for hypocalcemic patients one could argue for early empirical calcium administration in bleeding trauma patients to avoid hypocalcemia and promote the priming effect in platelets [18]. However, when supplementing calcium, the risk of hypercalcemia should be considered. In this analysis, hypercalcemia on arrival at the ED was associated with worst outcomes.

Increasing iCa2+ levels were associated with an increasing incidence of coagulopathy, transfusion requirement, and mortality (6 h, 24 h, and in-hospital). The cumulative survival at 30d was even lower compared to hypocalcemia, with survival differences again being mostly related to differences in early mortality within the first day. Mortality within the first day was more than double in hypercalcemic compared to normocalcemic patients (13.2% vs. 6.0%). Likewise, MacKay et al. [16] also identified an increased mortality in hypercalcemic trauma patients during trauma resuscitation with high- volume transfusion.

However, the independent association of hypercalcemia with coagulopathy and transfusion conflicts with the theory of calcium-induced platelet priming and thus requires different explanations. It is known that in critical ill patients the fraction of free ionized calcium changes due to alterations in serum protein concentrations and acid–base balance disturbances [5]. In the present analysis, acidosis was indeed much more common in the hypercalcemia group (40.1%). Acidosis increases the fraction of unbound ionized calcium, as hydrogen ions will compete with free calcium ions for protein binding sites [33, 34]. Moreover, acute metabolic and respiratory acidosis will stimulate parathyroid hormone secretion, leading to an increase in calcium concentrations [35]. Therefore, trauma-induced acid–base disturbances will affect ionized calcium levels. On the other hand, appropriate resuscitation and/or mechanical ventilation will restore the acid–base balance with an inverse effect on ionized calcium levels. Therefore, a drop in iCa2 + can be expected when adequate resuscitation is applied in acidotic trauma patients. This might explain the finding that 11.2% of hypercalcemic patients still received calcium supplementation during resuscitation in the ED.

Our findings do not support routine early empiric calcium supplementation. There is no simplistic linear relation between iCa2+ levels and outcome. Both hypo- and hypercalcemia have an independent association with coagulopathy and transfusion requirement. Despite a similar parabolic relation with unadjusted mortality, this parabolic relationship could not be demonstrated for 24 h or in- hospital mortality in a multivariable logistic regression model. This finding demonstrates that there is also no evidence to assume that early strict management of iCa2+ levels, with aggressive correction of both hypo- and hypercalcemia, has a beneficial effect on survival.

The injury itself, the accompanying shock, and subsequent resuscitation will all affect the iCa2+ levels after trauma. Therefore, iCa2+ levels might be a much more dynamic concept and the importance during trauma resuscitation might be much more complex than is believed to date.

### Limitations

A retrospective observational study design comes with its inherent limitations. Associations can be determined but causality cannot be proven, therefore result should be interpreted with caution and in their clinical context.

Additionally, some specific limitations for this study need to be addressed.

First, measurement errors cannot be excluded. Moreover, the exact timing of the measurement after arrival may slightly differ between samples. However, obtaining the first blood samples before extensive resuscitation is considered common practice and the large sample size used in this analysis will likely compensate for possible errors.

Second, the exclusion of patients with missing iCa2+ levels could induce some bias in generalizing the results to the entire trauma population. No information was available whether this missing value was because of not measuring a blood gas sample or if this was a registration error. This excluded group had a slightly lower injury severity. Likely, the threshold to perform a blood gas analysis on arrival will be higher in less severely injured patients. Nevertheless, we believe that the sample we present in this study is representative for the overall major trauma population.

Third, data about prehospital transfusion was not available in the dataset. However, in a recent survey by the European Society of Anaesthesiology only 6% of respondents from German Helicopter Emergency Medical Services (HEMS) had access to pre-hospital blood products. Moreover, in the overall European cohort, the majority of services with blood products available only used them very rarely, also including non-trauma cases [26, 36]. Therefore, prehospital blood transfusion was not considered as a routine practice during our study period, and any incidental use is unlikely to change the conclusion of our study.

Fourth, base excess was used as a marker of metabolic acidosis. As no pH or pCO_2_ levels were available, the contribution of respiratory acidosis or alkalosis could not be assessed.

## Conclusions

Ionized calcium levels in major trauma patients upon arrival at the ED have a parabolic relationship with outcome. In this multicentre analysis of 30 183 patients with major trauma, both hypo- and hypercalcemia were associated with an increased incidence of coagulopathy, need for transfusion, and mortality (6 h, 24 h, in-hospital). In a multivariable logistic regression model, this parabolic relationship with coagulopathy, transfusion requirement, and 6 h mortality was confirmed. However, no parabolic or linear relationship with 24 h and in-hospital mortality could be demonstrated.

This study shows no convincing evidence to support routine empirical administration of calcium. Further research is needed to confirm whether altered ionized calcium levels are more a reflection of severity of injury and accompanying physiological derangements, rather than an individual parameter that needs to be corrected as such. To better understand the dynamic alterations in iCa2+ levels, prospective trials should monitor iCa2+ levels at different time points after injury, in relation to acid–base status and resuscitation strategies.

## Supplementary Information

**Additional file 1**. Description of the TraumaRegister DGU^®^.

**Additional file 2**. Distribution and accompanying outcome parameters of ionized calcium levels in adult major trauma patients.

**Additional file 3**. Demographics and clinical characteristics for different ranges of ionized calcium levels.

## Data Availability

The sensitive data presented in this study are available from a third party, the AUC (Academy for Trauma Surgery), which is the holder of the data of the TraumaRegister DGU^®^. The data protection concept of TraumaRegister DGU^®^ includes that no raw data are available for external use. More information is available from: AUC—Akademie der Unfallchirurgie GmbH, Emil-Riedel-Straße 5, 80538 München, Deutschland, Email: support-tr@auc-online.de.

## Abbreviations

iCa2 +: Ionized calcium
TIC: Trauma-induced coagulopathy
ED: Emergency department
ICU: Intensive care unit
AIS: Abbreviated injury scale
PTT: Partial thromboplastin time
INR: International normalized ratio
SD: Standard deviation
IQR: Interquartile range
ISS: Injury severity score
GCS: Glasgow coma scale
RISC II: Revised injury severity classification II
OR: Odds ratio
SI: Shock index
HEMS: Helicopter Emergency Medical Service
