# Supplementary material for: Trauma-induced disturbances in ionized calcium levels correlate parabolically with coagulopathy, transfusion, and mortality: a multicentre cohort analysis from the TraumaRegister DGU®
Source: Crit Care. 2023 Jul 6;27:267. doi: 10.1186/s13054-023-04541-3 (PMC10324195; doi:10.1186/s13054-023-04541-3)
Supplement: Supplementary file 1 — Additional file 1. Description of the TraumaRegister DGU®. [file 13054_2023_4541_MOESM1_ESM.pdf]

ADDITIONAL FILE 1: Description of the TraumaRegister DGU®

The TraumaRegister DGU® of the German Trauma Society (Deutsche Gesellschaft für Unfallchirurgie, DGU) was founded in 1993. The aim of this multi-centre database is a pseudonymized and standardized documentation of severely injured patients.

Data are collected prospectively in four consecutive time phases from the site of the accident until discharge from hospital: A) Pre-hospital phase, B) Emergency room and initial surgery, C) Intensive care unit (ICU) and D) Discharge. The documentation includes detailed information on demographics, injury pattern, comorbidities, pre- and in-hospital management, course on intensive care unit, relevant laboratory findings including data on transfusion and outcome of each individual. The inclusion criterion is admission to hospital via emergency department with subsequent ICU/ICM care or reach the hospital with vital signs and die before admission to ICU.

The infrastructure for documentation, data management, and data analysis is provided by AUC - Academy for Trauma Surgery (AUC - Akademie der Unfallchirurgie GmbH), a company affiliated to the German Trauma Society. The scientific leadership is provided by the Committee on Emergency Medicine, Intensive Care and Trauma Management (Sektion NIS) of the German Trauma Society. The participating hospitals submit their data pseudonymized into a central database via a web-based application. Scientific data ana- lysis is approved according to a peer review procedure laid down in the publication guideline of TraumaRegister DGU®.

The participating hospitals are primarily located in Germany (90%), but a rising number of hospitals of other countries contribute data as well (at the moment from Austria, Belgium, Finland, Luxembourg, Slovenia, Switzerland, The Netherlands, and the United Arab Emirates). Currently, over 30 000 cases from almost 700 hospitals are entered into the database per year.

Participation in TraumaRegister DGU® is voluntary. For hospitals associated with TraumaNetzwerk DGU®, however, the entry of at least a basic data set is obligatory for reasons of quality assurance.
