# Supplementary material for: Trauma-induced disturbances in ionized calcium levels correlate parabolically with coagulopathy, transfusion, and mortality: a multicentre cohort analysis from the TraumaRegister DGU®
Source: Crit Care. 2023 Jul 6;27:267. doi: 10.1186/s13054-023-04541-3 (PMC10324195; doi:10.1186/s13054-023-04541-3)
Supplement: Supplementary file 2 — Additional file 2. Distribution and accompanying outcome parameters of ionized calcium levels in adult major trauma patients. [file 13054_2023_4541_MOESM2_ESM.pdf]

ADDITIONAL FILE 2: Distribution and accompanying outcome parameters of ionized calcium levels in adult major trauma patients.

NAME	REFERENCE RANGE	INCIDENCE		IN-HOSPITAL MORTALITY		6h MORTALITY		24h MORTALITY		COAGULO-PATHY		TRANSFUSION	
		n	%	n	%	n	%	n	%	n	%	n	%
<0.9	<0.90	240	0.8	87	36.3	33	13.8	55	22.9	117	48.8	114	47.5
0.9	0.90 – 0.99	307	1.0	94	30.6	15	4.9	53	17.3	115	37.5	115	37.5
1.0	1.00 – 1.09	3435	11.4	662	19.3	81	2.4	305	8.9	641	18.7	614	17.9
1.1	1.10 – 1.19	15730	52.1	2181	13.9	264	1.7	950	6.0	2105	13.4	1802	11.5
1.2	1.20 – 1.29	9508	31.5	1251	13.2	190	2.0	561	5.9	1247	13.1	1145	12.0
1.3	1.30 – 1.39	745	2.5	170	22.8	42	5.6	92	12.3	218	29.3	190	25.5
≥1.4	≥1.40	218	0.7	55	25.2	20	9.2	35	16.1	89	40.8	62	28.4
TOTAL		30183	100	4500	14.9	645	2.1	2051	6.8	4532	15.0	4042	13.4
