# Supplementary material for: Trauma-induced disturbances in ionized calcium levels correlate parabolically with coagulopathy, transfusion, and mortality: a multicentre cohort analysis from the TraumaRegister DGU®
Source: Crit Care. 2023 Jul 6;27:267. doi: 10.1186/s13054-023-04541-3 (PMC10324195; doi:10.1186/s13054-023-04541-3)
Supplement: Supplementary file 3 — Additional file 3. Demographics and clinical characteristics for different ranges of ionized calcium levels. [file 13054_2023_4541_MOESM3_ESM.pdf]

ADDITIONAL FILE 3: Demographics and clinical characteristics for different ranges of ionized calcium levels.

VARIABLES	OVERALL	HYPOcalcemia (<1.10mmol/L)	NORMOcalcemia (1.10-1.29mmol/L)	HYPERcalcemia (≥1.30mmol/L)
**Total (%)**	**30183 (100)**	**3982 (13.2)**	**25238 (83.6)**	**963 (3.2)**
**Patient**				
Sex, male (%)	21655 (71.7)	2933 (71.1)	18160 (72.0)	662 (68.7)
Age, median (IQR)	54 (35-70)	54 (37-70)	54 (35-70)	55 (33-72)
Anticoagulation therapy before accident (%)	4700 (15.6)	595 (14.9)	3953 (15.7)	152 (15.8)
**Accident Mechanism**				
Blunt (%)	27872 (95.5)	3563 (93.4)	23436 (95.9)	873 (94.1)
Penetrating (%)	1309 (4.5)	253 (6.6)	1001 (4.1)	55 (5.9)
**Injury Severity**				
ISS , median (IQR)	21 (14-29)	22 (16-29)	20 (14-29)	22 (16-29)
AIS≥3 head (%)	14527 (48.1)	2084 (52.3)	11997 (47.5)	446 (46.3)
Isolated AIS≥3 head (%)	4677 (15.5)	733 (18.4)	3821 (15.1)	123 (12.8)
AIS≥3 thorax (%)	14784 (49.0)	1877 (47.1)	12404 (49.1)	503 (52.2)
AIS≥3 abdomen (%)	3983 (13.2)	614 (15.4)	3205 (12.7)	164 (17.0)
AIS≥3 extremities (%)	9204 (30.5)	1253 (31.5)	7594 (30.1)	357 (37.1)
**Pre- clinical vital signs**				
sBP ≤90 mmHg (%)	2791 (10.5)	512 (15.0)	2125 (9.5)	154 (18.2)
Shock Index ≥1 (%)	3478 (13.5)	587 (18.1)	2746 (12.7)	145 (18.1)
GCS ≤8 (%)	7032 (24.7)	1237 (33.1)	5532 (23.2)	263 (28.7)
**Pre- clinical therapy**				
Volume administration >1000ml (%)	4189 (15.2)	548 (15.5)	3456 (14.9)	185 (20.8)
Intubation (%)	9757 (33.1)	1518 (39.5)	7846 (31.8)	393 (41.4)
TXA (%)	4430 (15.0)	672 (17.5)	3593 (14.6)	165 (17.4)
CPR (%)	1049 (3.6)	199 (5.2)	760 (3.1)	90 (9.5)
Pleural decompression (%)	1184 (4.0)	192 (5.0)	919 (3.7)	73 (7.7)
Cathecholamines (%)	3269 (11.1)	567 (14.7)	2514 (10.2)	188 (19.8)
**Vital signs on admission**				
sBP ≤90 mmHg (%)	3072 (10.6)	588 (15.5)	2301 (9.4)	183 (19.8)
Shock Index ≥1 (%)	3860 (13.5)	691 (18.7)	2976 (12.4)	193 (21.7)
Temperature <35°C (%)	2089 (10.8)	386 (16.1)	1600 (9.8)	103 (16.6)
**Laboratory values on admission**				
Hb, g/dL, mean (SD)	12.9 (2.2)	12.3 (2.6)	13.0 (2.1)	11.7 (2.8)
INR, mean (SD)	1.2 (0.6)	1.3 (0.7)	1.2 (0.5)	1.4 (0.9)
PT, %, mean (SD)	84.6 (21.9)	80.6 (24.4)	85.6 (21.2)	75.1 (25.3)
Platelets, x10^9/L, mean (SD)	224 (78)	215 (82)	227 (77)	205 (81)
Coagulopathy (%)	4532 (15.0)	873 (21.9)	3352 (13.3)	307 (31.9)

Base Excess, mean (SD)	-2.2 (4.8)	-3.6 (6.1)	-1.8 (4.3)	-6.2 (7.3)
Acidosis, BE < -6 (%)	4555 (15.2)	997 (25.3)	3180 (12.7)	378 (40.1)
**Transfusion prior to ICU admission**				
Time to transfusion RBC, min, median (IQR)	68 (25-121)	44 (17-101)	76 (30-128)	49 (16-101)
Transfusion (%)	4042 (13.4)	843 (21.2)	2947 (11.7)	252 (26.2)
RBC (units) if transfused, median (IQR)	4 (2-6)	4 (2-8)	3 (2-6)	4 (2-8)
FFP (units) if transfused, median (IQR)	2 (0-5)	2 (0-6)	1 (0-4)	1 (0-6)
Platelets (units) if transfused, median (IQR)	0 (0-0)	0 (0-1)	0 (0-0)	0 (0-0)
Massive transfusion ≥10 RBC (%)	528 (1.7)	149 (3.7)	335 (1.3)	44 (4.6)
**Procoagulant therapy prior to ICU admission**				
Ca2+ supplement (%)	1593 (5.6)	372 (10.4)	1120 (4.7)	101 (11.2)
Ca2+ supplement if transfused (%)	1179 (30.5)	288 (36.1)	806 (28.6)	85 (34.7)
TXA (%)	5996 (21.2)	1032 (28.9)	4693 (19.7)	271 (29.9)
**Length-of-stay**				
ICU days, median (IQR)	3 (1-11)	4 (2-12)	3 (1-10)	4 (1-13)
Hospital days, median (IQR)	14 (7-24)	14 (6-26)	14 (7-24)	14 (6-25)
**Prognosis**				
Expected mortality RISC II %, mean	14.1	18.7	13.1	23.2
Compared to measured mortality	-0.8	-2.5	-0.5	-0.2
**Mortality**				
In- hospital mortality (%)	4500 (14.9)	843 (21.2)	3432 (13.6)	225 (23.4)
Mortality in emergency room (%)	293 (1.0)	62 (1.6)	192 (0.8)	39 (4.2)
Mortality <1h (%)	131 (0.4)	30 (0.8)	82 (0.3)	19 (2.0)
Mortality <24h (%)	2051 (6.8)	413 (10.4)	1511 (6.0)	127 (13.2)
**Cause of death**				
Haemorrhage (%)	283 (6.4)	88 (10.7)	166 (4.9)	29 (13.1)
Traumatic Brain Injury (%)	2653 (60.4)	451 (55.1)	2098 (62.5)	104 (47.1)
Organ failure (%)	1039 (23.6)	205 (25.0)	761 (22.7)	73 (33.0)
Other (%)	421 (9.6)	75 (9.2)	331 (9.9)	15 (6.8)

ISS: Injury Severity Score - AIS: Abbreviated Injury Scale - sBP: Systolic Blood Pressure - GCS: Glasgow Coma Scale - TXA: Tranexamic Acid - CPR: Cardiopulmonary Resuscitation - Hb: Haemoglobin - INR: International Normalized Ratio - PT: Prothrombin Time - BE: Base Excess - PRC: Packed Red Cells - FFP: Fresh Frozen Plasma - Ca2+: Calcium - ICU: Intensive Care Unit - RISC-II: Revised Injury Severity Classification , version II - IQR: Interquartile Range - SD: Standard Deviation
